# Curcumin/Turmeric Supplementation on Glycemic Control in Adults With Prediabetes and Type 2 Diabetes: A Systematic Review and Dose–Response Meta‐Analysis

**DOI:** 10.1002/fsn3.71748

**Published:** 2026-04-16

**Authors:** Hossein Bahari, Mostafa Shahraki Jazinaki, Zahra Asadi, Haniyeh Golafrouz

**Affiliations:** ^1^ Department of Nutrition, Faculty of Medicine Mashhad University of Medical Sciences Mashhad Iran; ^2^ Student Research Committee Mashhad University of Medical Sciences Mashhad Iran; ^3^ Student Research Committee Shiraz University of Medical Sciences Shiraz Iran; ^4^ Rajaei Cardiovascular Medical and Research Center Iran University of Medical Sciences Tehran Iran

**Keywords:** curcumin, diabetes, insulin resistance, meta‐analysis, prediabetes, turmeric

## Abstract

Curcumin and turmeric have demonstrated potential hypoglycemic properties in preclinical studies, but findings from human randomized controlled trials (RCTs) in prediabetes and type 2 diabetes (T2D) are inconsistent. PubMed, Scopus, and Web of Science ISI databases were comprehensively searched until August 2025 (25/08/2025) to find eligible RCTs. Overall effect sizes were estimated based on the random‐effect models and presented as weighted mean differences (WMD) with 95% confidence intervals (95% CI). All analyses were performed using version 17.0 (Stata Corp., College Station, TX, USA). Thirty‐four eligible RCTs (39 treatment arms) were included in this review. Curcumin/turmeric supplementation significantly reduced fasting blood glucose (FBG) (WMD = −10.15 mg/dL; 95% CI: −12.59, −7.72), hemoglobin A1c (HbA1c) (WMD = −0.32%; 95% CI: −0.43, −0.21), fasting insulin (WMD = −0.69 μU/ml; 95% CI: −1.27, −0.12), homeostatic model assessment of insulin resistance (HOMA‐IR) (WMD = −0.46; 95% CI: −0.60, −0.32), and oral glucose tolerance test (OGTT) (WMD = −11.53 mg/dL; 95% CI: −22.68, −0.44) compared to control groups. However, no significant alteration in homeostatic model assessment of *β*‐cell function (HOMA‐B) levels was detected, followed by Curcumin or turmeric supplementation in comparison to the control groups. Subgroup analyses indicated that efficacy was modified by health status (T2D vs. prediabetes), dosage (≥ 1 g/day more effective), and formulation. This meta‐analysis revealed that curcumin/turmeric supplementation may improve glycemic control in individuals with prediabetes or T2D. Despite positive findings, significant heterogeneity across studies necessitates caution. Furthermore, future high‐quality RCTs are required to reach a firm conclusion.

## Graphical abstract

This meta‐analysis of 34 randomized controlled trials shows that curcumin/turmeric supplementation significantly improves glycemic control in prediabetes and type 2 diabetes. Key reductions were observed in fasting blood glucose (−10.15 mg/dL), HbA1c (−0.32%), fasting insulin, and HOMA‐IR. Efficacy was influenced by dosage (≥ 1 g/day more effective), formulation, and health status. No significant effect was found on HOMA‐B. Findings support curcumin as a potential adjunct therapy, though heterogeneity warrants cautious interpretation.

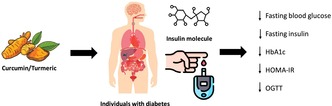

## 1 Introduction

The global prevalence of diabetes mellitus, particularly type 2 diabetes (T2D), and its precursor state, prediabetes, has reached epidemic proportions, posing a significant threat to public health systems worldwide (Sun et al. 2022). This metabolic disorder is characterized by chronic hyperglycemia resulting from defects in insulin secretion, insulin action, or both, and is a leading cause of microvascular and macrovascular complications, including neuropathy, nephropathy, retinopathy, and cardiovascular disease (Elsayed et al. 2025). Prediabetes, defined by impaired fasting glucose (IFG) or impaired glucose tolerance (IGT), represents a critical window for intervention to halt or delay the progression to overt T2D (Tabák et al. 2012).

Current management strategies for hyperglycemia primarily involve lifestyle modifications and pharmacotherapeutic agents such as metformin, sulfonylureas, and insulin (Davies et al. 2022). However, these conventional treatments can be associated with side effects, high costs, and inadequate efficacy in some patients, driving the search for complementary and alternative therapeutic approaches (Pang et al. 2019). In this context, renewed attention has been directed toward natural products, traditionally recognized for their therapeutic properties, to evaluate their role in the prevention and management of T2D (Li et al. 2025; Mkhize et al. 2025; Mokgalaboni and Phoswa 2023; Sknepnek et al. 2025).

Curcumin, a naturally occurring polyphenol derived from the rhizome of the turmeric plant ( *Curcuma longa* ), is one such compound. It has been used for centuries in traditional medicine and is renowned for its potent anti‐inflammatory, antioxidant, and antimicrobial properties (El‐Saadony et al. 2023). A growing body of preclinical evidence suggests that curcumin and its formulations can improve glycemic control through multiple mechanisms. These include enhancing insulin sensitivity in peripheral tissues, preserving pancreatic *β*‐cell function, reducing hepatic gluconeogenesis, and ameliorating chronic inflammation and oxidative stress, which are key pathophysiological features of insulin resistance and T2D (Bhatti et al. 2022; Liu et al. 2025; Marton, Pescinini‐e‐Salzedas, et al. 2021; Marton, Pescinini, et al. 2021).

Numerous randomized controlled trials (RCTs) have investigated the effects of curcumin and turmeric supplementation on various markers of glycemic control, such as fasting blood glucose (FBG), glycated hemoglobin (HbA1c), insulin levels, and homeostatic model assessments for insulin resistance (HOMA‐IR) and beta‐cell function (HOMA‐B) (Adab et al. 2019; Maithili Karpaga Selvi et al. 2015; Rahimi et al. 2016). However, the results from these individual studies have been inconsistent. This inconsistency may be attributed to variations in study design, participant characteristics (e.g., prediabetes vs. diabetes), the dosage and formulation of curcumin used (e.g., plain curcumin, curcuminoids, nano‐curcumin, or bioavailability‐enhanced formulations with piperine), and the duration of intervention (Pathomwichaiwat et al. 2023; Pivari et al. 2019). In the previous meta‐analyses, a significant reduction in FBG and HbA1c levels was reported, followed by curcumin/turmeric supplementation compared to the placebo in adults with T2D. However, in these meta‐analyses, the impacts of curcumin/turmeric supplementation on other glycemic control markers were not investigated. Also, to our best knowledge, no systematic review and meta‐analysis have assessed the effects of curcumin/turmeric supplementation on glycemic control, involving both prediabetics and T2D individuals. In addition, while previous meta‐analyses have attempted to synthesize this evidence, the rapidly expanding number of new clinical trials, including recent high‐quality studies, necessitates a comprehensive re‐quantifying and synthesis (El‐Rakabawy et al. 2025; Ghazaee et al. 2025; Mansour et al. 2025; Yaikwawong et al. 2025). Therefore, this systematic review and dose–response meta‐analysis aims to comprehensively evaluate and quantify the effects of curcumin/turmeric supplementation on glycemic control in adults with prediabetes and type.

## 2 Methods

### 2.1 Protocol and Registration

The protocol for this systematic review and meta‐analysis was prospectively registered with the International Prospective Register of Systematic Reviews (PROSPERO) under registration number CRD420251146630. The review was conducted and reported in accordance with the Preferred Reporting Items for Systematic Reviews and Meta‐Analyses (PRISMA) statement (Moher, Liberati, Tetzlaff, Altman, and Group* 2009; Page et al. 2021).

### 2.2 Search Strategy

A systematic search of the following electronic databases was conducted from inception until August 2025 (25/08/2025): PubMed, Scopus, and Web of Science. The search strategy utilized a combination of Medical Subject Headings (MeSH) terms and other related keywords as follows: (“curcumin” OR “turmeric” OR “curcuminoid*”) AND (“diabetes” OR “prediabetes”) AND (“randomized controlled trial” OR “RCT” OR “parallel” OR “cross‐over” OR “clinical trial”). The details of the search strategy used in each database are provided in Table S1. The reference lists of all eligible studies and relevant review articles were checked to identify any additional eligible studies. Also, the Google Scholar search engine was manually searched.

### 2.3 Eligibility Criteria

Found studies were assessed for inclusion in this systematic review based on the following PICOS (Participants, Intervention, Comparison, Outcomes, Study design) criteria:

- Participants: Adult human subjects (≥ 18 years) diagnosed with prediabetes (impaired fasting glucose, impaired glucose tolerance) or T2D, as defined by the study authors or standard guidelines (e.g., ADA, WHO).
- Intervention: Oral supplementation with any form of curcumin or turmeric, including but not limited to: curcumin, curcuminoids, turmeric powder, nano‐curcumin, and bioavailability‐enhanced formulations (e.g., combined with piperine, phospholipids, etc.). Co‐supplementation was allowed only if the control group received the same additional supplement.
- Comparison: Placebo or any active control (e.g., conventional therapy, metformin).
- Outcomes: Glycemic control outcomes, including FBG, HbA1c, fasting insulin, HOMA‐IR, HOMA‐B, and OGTT.
- Study Design: RCTs, either parallel or crossover design.

Records with the following features were excluded from this review: (a) in vitro or animal studies, (b) studies on type 1 or gestational diabetes, (c) observational studies such as case–control, cohort, cross‐sectional, etc., (d) 4 review articles, editorials, and conference abstracts; and (e) studies that used curcumin as part of a multi‐component intervention where its effect could not be isolated (e.g., in a complex herbal mixture), (f) or trials that did not report mean changes of glycemic control outcomes followed by intervention.

### 2.4 Study Selection and Data Extraction

Two investigators (Z.A. and M.S.J.) independently screened all identified records according to their titles and abstracts. The full texts of potentially relevant studies were then retrieved and assessed for eligibility based on the predefined criteria. Any disagreements between the reviewers were resolved through discussion or by consultation with a third reviewer (H.B.).

Data from the included studies were extracted independently by two reviewers (Z.A. and M.S.J.) using a pre‐piloted, standardized data extraction form. The following information was collected: first author's name, publication year, country of origin, study design, participant characteristics (sample size, age, gender, health status, BMI), details of the intervention and control groups (type of intervention, dosage, and duration), and the means and standard deviations (SDs) of changes in outcomes of interest. For studies with more than one intervention arm, each relevant arm was extracted separately and treated as an independent “study arm” in the analysis.

### 2.5 Risk of Bias Assessment

The risk of bias for each included RCT was assessed independently by two reviewers (Z.A. and M.S.J.) using the revised Cochrane risk‐of‐bias tool for randomized trials (RoB 2) (Sterne et al. 2019). The tool evaluates bias across five domains: (1) bias arising from the randomization process, (2) bias due to deviations from intended interventions, (3) bias due to missing outcome data, (4) bias in measurement of the outcome, and (5) bias in selection of the reported result. Each study was judged to have a “low risk of bias,” “some concerns,” or “high risk of bias” for each domain. An overall risk of bias judgment for each study was then generated based on the domain‐level judgments.

### 2.6 Statistical Analysis

In this meta‐analysis, all analyses were performed using STATA software version 17 (Stata Corp, College Station, TX, USA). The overall effect size was calculated based on the random‐effects model by using the mean and SD of change for each outcome. The pooled effect size was expressed as the weighted mean difference (WMD) with a 95% confidence interval (95% CI). Heterogeneity among the included studies was assessed using Cochran's Q test (with a significance level of *p* < 0.10) and the *I* ^2^ statistic. An *I* ^2^ value greater than 50% was considered to represent substantial heterogeneity.

Pre‐specified subgroup analyses were conducted to explore potential sources of heterogeneity, including: health status (prediabetes vs. T2D), intervention dosage (< 1000 vs. ≥ 1000 mg/day), intervention formulation (unformulated curcumin/curcuminoids, nano‐curcumin, and bioavailability‐enhanced), intervention duration (< 12 vs. ≥ 12 weeks), and baseline BMI category (normal, overweight, obese). Meta‐regression analysis was performed to examine the potential linear relationship between the continuous variables (dosage and duration) and the change in each outcome level. A two‐stage, random‐effects dose–response analysis was performed using fractional polynomial modeling to explore potential non‐linear relationships between the dosage/duration of curcumin supplementation and the changes in outcome measures (Xu and Doi 2018). Sensitivity analysis was conducted by sequentially removing each study from the analysis to evaluate the stability of the pooled results (Tobias 1999). Publication bias was assessed visually through inspection of funnel plots and formally using Egger's regression test, with a *p*‐value < 0.05 indicating potential small‐study effects (Egger et al. 1997).

### 2.7 Certainty of Evidence

The certainty of evidence for each outcome was assessed using the Grading of Recommendations, Assessment, Development, and Evaluations (GRADE) approach, which assessed limitations in five domains of risk of bias, inconsistency, indirectness, imprecision, and publication bias (Guyatt et al. 2011). Overall evidence certainty was graded into four levels, including very low, low, moderate, and high.

## 3 Results

### 3.1 Study Selection

**FIGURE 1:**
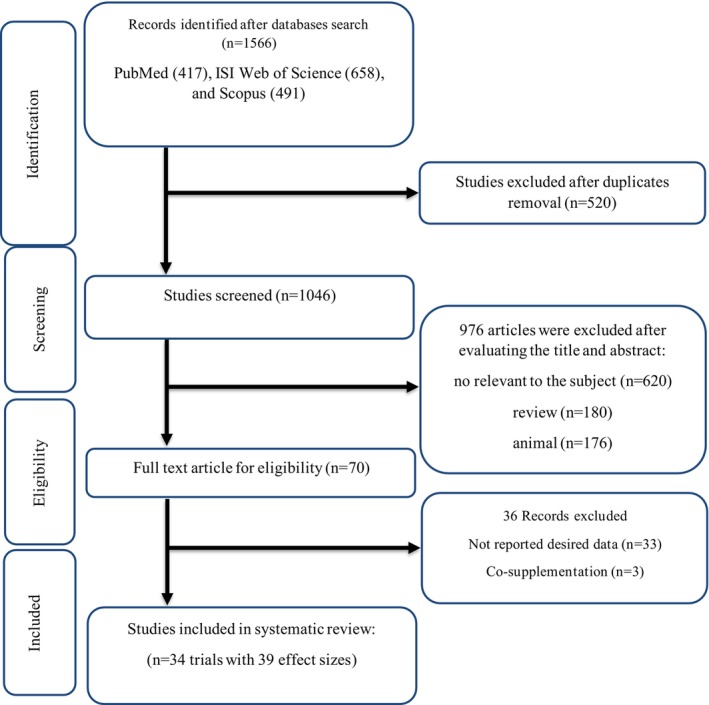
Flow chart of study selection for inclusion trials in the systematic review.

Out of the 1566 found records, 520 duplicated cases were deleted. In the next step, 1046 papers were screened based on their abstracts and titles, resulting in the exclusion of 976 records due to not being relevant to the subject (*n* = 620), animal studies (*n* = 176), and review articles (*n* = 180). The full text of 70 remaining studies was accurately read, which led to the exclusion of 36 cases due to co‐supplementation (*n* = 3) and not reporting required data (*n* = 33). Finally, 34 studies with 39 treatment arms were eligible for inclusion in this systematic review and meta‐analysis (Adab et al. 2019; Asadi et al. 2019; Asghari et al. 2024; Chuengsamarn et al. 2012, 2014; Cicero et al. 2020; Darmian et al. 2021; El‐Rakabawy et al. 2025; Funamoto et al. 2019; Ghazaee et al. 2025; Hodaei et al. 2019; Hosseini et al. 2024; Jiménez‐Osorio et al. 2016; Karandish et al. 2021; Khajehdehi et al. 2011; Lamichhane et al. 2025; Maithili Karpaga Selvi et al. 2015; Mansour et al. 2025; Mokhtari et al. 2021; Na et al. 2013; Nada et al. 2025; Neta et al. 2021; Panahi et al. 2018; Rahimi et al. 2016; Shafabakhsh et al. 2020; Sousa et al. 2021; Thota et al. 2019, 2020; Uchio et al. 2024; Usharani et al. 2008; Vanaie et al. 2019; Yaikwawong et al. 2024, 2025; Zamani and Rezagholizadeh 2021). The PRISMA flow chart of the study selection process is shown in Figure 1.

### 3.2 Study Characteristics

Eligible studies were published between 2008 (Usharani et al. 2008) and 2025 (El‐Rakabawy et al. 2025; Ghazaee et al. 2025; Lamichhane et al. 2025; Mansour et al. 2025; Nada et al. 2025; Yaikwawong et al. 2025). India (Maithili Karpaga Selvi et al. 2015; Usharani et al. 2008), Iran (Adab et al. 2019; Asadi et al. 2019; Asghari et al. 2024; Darmian et al. 2021; Ghazaee et al. 2025; Hodaei et al. 2019; Hosseini et al. 2024; Karandish et al. 2021; Khajehdehi et al. 2011; Mansour et al. 2025; Mokhtari et al. 2021; Panahi et al. 2018; Rahimi et al. 2016; Shafabakhsh et al. 2020; Vanaie et al. 2019; Zamani and Rezagholizadeh 2021), Thailand (Chuengsamarn et al. 2012, 2014; Yaikwawong et al. 2024, 2025), China (Na et al. 2013), Mexico (Jiménez‐Osorio et al. 2016), Egypt (El‐Rakabawy et al. 2025; Nada et al. 2025), Australia (Thota et al. 2019, 2020), Japan (Funamoto et al. 2019; Uchio et al. 2024), Italy (Cicero et al. 2020), Brazil (Neta et al. 2021; Sousa et al. 2021), and the USA (Lamichhane et al. 2025), were the origin countries of studies. Among the 34 eligible papers, two were conducted exclusively on females (Darmian et al. 2021; Zamani and Rezagholizadeh 2021), and one on exclusively males (Maithili Karpaga Selvi et al. 2015), while the rest of the included trials involved both genders. The sample sizes of the included studies were varied from 20 (Zamani and Rezagholizadeh 2021) to 235 participants (Chuengsamarn et al. 2012). Mean age and mean BMI in the populations of included effect sizes ranged between 35.5 (Karandish et al. 2021) and 69.5 years old (Funamoto et al. 2019), and 24.3 (Usharani et al. 2008) and 32.7 kg/m^2^ (Nada et al. 2025), respectively. However, one of the included trials did not report the mean age (Yaikwawong et al. 2024), and two did not report the mean BMI of their populations (Khajehdehi et al. 2011; Vanaie et al. 2019), respectively. The durations and dosages of intervention were between 4 (Maithili Karpaga Selvi et al. 2015) and 48 weeks (Yaikwawong et al. 2024), and 80 (Asghari et al. 2024; Lamichhane et al. 2025; Mansour et al. 2025; Zamani and Rezagholizadeh 2021) and 2100 g/day (Adab et al. 2019; Darmian et al. 2021), respectively. The intervention types included curcumin (Asghari et al. 2024; El‐Rakabawy et al. 2025; Karandish et al. 2021; Lamichhane et al. 2025; Vanaie et al. 2019; Yaikwawong et al. 2024, 2025; Zamani and Rezagholizadeh 2021), turmeric (Adab et al. 2019; Darmian et al. 2021; Jiménez‐Osorio et al. 2016; Khajehdehi et al. 2011; Maithili Karpaga Selvi et al. 2015), curcuminoids (Chuengsamarn et al. 2012, 2014; Na et al. 2013; Usharani et al. 2008), nano‐curcumin (Asadi et al. 2019; Mansour et al. 2025; Mokhtari et al. 2021; Rahimi et al. 2016; Shafabakhsh et al. 2020), Meriv (Thota et al. 2019, 2020), theracurmin (Funamoto et al. 2019), curcumin combined with piperine (Cicero et al. 2020; Ghazaee et al. 2025; Hosseini et al. 2024; Neta et al. 2021; Panahi et al. 2018; Sousa et al. 2021), curcuminoids combined with turmeric oil (Hodaei et al. 2019), *curcuma longa* extract (Uchio et al. 2024), and curcumin combined with black pepper (Nada et al. 2025). In the control groups, one study used conventional therapy (El‐Rakabawy et al. 2025), another zinc with lifestyle modification (Karandish et al. 2021), one administered metformin (Maithili Karpaga Selvi et al. 2015), another involved training (Zamani and Rezagholizadeh 2021), and one received nothing (Zamani and Rezagholizadeh 2021), while in the rest of the eligible studies, the control groups received a placebo. Among the included studies, seven were conducted on individuals with prediabetes (Cicero et al. 2020; Ghazaee et al. 2025; Karandish et al. 2021; Lamichhane et al. 2025; Thota et al. 2019, 2020; Uchio et al. 2024), while the other included trials performed interventions on participants with T2D. The characteristics of eligible studies are summarized in Table 1.

**TABLE 1:** Characteristics of included studies in meta‐analysis.

Studies	Country	Study design	Participant	Sex	Sample size		Trial duration (Week)	Means Age		Means BMI		Intervention		
					IG	CG		IG	CG	IG	CG	Type	Dose (mg/day)	Control group
Usharani et al. 2008	India	R, DB, PC, Parallel	T2D	B	23	21	8	55.52	49.75	24.66	23.98	NCB‐02 (standardized curcuminoid capsule)	600	Placebo
Khajehdehi et al. 2011	Iran	R, DB, PC, Parallel	T2D nephropathy	B	20	20	8	52.9	52.6	NR	NR	Turmeric	1500	Placebo (starch)
Chuengsamarn et al. 2012	Thailand	R, DB, PC, Parallel	Prediabetic	B	116	119	36	56.95	57.93	26.66	26.62	Curcuminoids	1500	Placebo
Na et al. 2013	China	R, DB, PC, Parallel	Overweight/obese T2D	B	50	50	12	55.42	54.72	27.12	27.42	Curcuminoids	300	Placebo
Chuengsamarn et al. 2014	Thailand	R, DB, PC, Parallel	T2D	B	107	106	24	59.16	59.58	27.09	26.84	Curcuminoids	1500	Placebo (starch)
Maithili Karpaga Selvi et al. 2015	India	R, Open, CO, Parallel	T2D	M	30	30	4	47	46.8	23.4	24.1	Turmeric + metformin	2000	Metformin
Jiménez‐Osorio et al. 2016	Mexico	R, DB, PC, Parallel	Diabetic proteinuric CKD	B	28	23	8	55	56.2	29.7	27.9	Turmeric	320	Placebo (starch)
Rahimi et al. 2016	Iran	R, DB, PC, Parallel	T2D	B	35	35	12	56.34	60.95	26.92	27.27	Nano‐curcumin	80	Placebo
Panahi et al. 2018	Iran	R, DB, PC, Parallel	T2D	B	50	50	12	43	41	26	27	Curcuminoids + piperine	500	Placebo
Adab et al. 2019	Iran	R, DB, PC, Parallel	Hyperlipidemic T2D	B	36	39	8	54.76	55.66	28.98	28.82	Turmeric	2100	Placebo (corn starch)
Asadi et al. 2019	Iran	R, DB, PC, Parallel	T2D	B	40	40	8	53.3	54.6	31.1	30.8	Nano‐curcumin	80	Placebo (poly‐sorbate)
Funamoto et al. 2019	Japan	R, DB, PC, Parallel	IGT and NIDDM	B	15	18	24	70	69	24.9	25	Theracurmin (highly absorbable curcumin)	180	Placebo
Hodaei et al. 2019	Iran	R, DB, PC, Parallel	NIDDM	B	21	23	10	58	60	29.2	28.2	Curcumin capsule (curcuminoids+ turmeric oil)	1500	Placebo (cooked rice flour)
Thota et al. 2019 (a)	Australia	R, DB, PC, Parallel	Adults with high risk of T2D	B	15	16	12	55	50	30.9	31.9	Meriva + corn oil	1000	Placebo + corn oil
Thota et al. 2019 (b)	Australia	R, DB, PC, Parallel	Adults with high risk of T2D	B	16	17	12	57	58	31.7	30	Meriva + fish oil	1000	Placebo + fish oil
Vanaie et al. 2019	Iran	R, DB, PC, Parallel	Overt diabetic nephropathy	B	27	19	16	59	61	NR	NR	Curcumin	1500	Placebo
Cicero et al. 2020	Italy	R, DB, PC, Parallel	Overweight with suboptimal FPG	B	40	40	8	54	53	27.1	26.9	Phytosomal curcumin + piperine	400	Placebo
Shafabakhsh et al. 2020	Iran	R, DB, PC, Parallel	Patients With Diabetes on HD	B	26	27	12	58.3	56.2	27.9	27.1	Nano‐curcumin	80	Placebo
Thota et al. 2020	Australia	R, DB, PC, Parallel	Adults with High Risk of T2D and Alzheimer's Disease	B	14	15	12	54.5	50.4	30.2	32.3	Meriva	1000	Placebo
Sousa et al. 2021	Brazil	R, DB, PC, Parallel	T2D	B	33	28	16	63.2	61.9	29.66	28.58	Curcumin + piperine	500	Placebo (carboxymethyl cellulose)
Darmian et al. 2021 (a)	Iran	R, SB, PC, Parallel	Middle‐Aged Females with T2D and Hyperlipidemia	F	11	10	8	44.33	44.22	29.3	29.02	Turmeric	2100	Placebo
Darmian et al. 2021 (b)	Iran	R, SB, PC, Parallel	Middle‐Aged Females with T2D and Hyperlipidemia	F	11	10	8	43.02	42.13	28.15	28.12	Turmeric + Aerobic training	2100	Placebo + Aerobic training
Karandish et al. 2021 (a)	Iran	R, DB, PC, Parallel	Overweight or obese prediabetic	B	21	20	12	36.95	34.19	30.46	30.97	Curcumin + lifestyle modification	500	Placebo + lifestyle modification
Karandish et al. 2021 (b)	Iran	R, DB, PC, Parallel	Overweight or obese prediabetic	B	20	21	12	34.48	38.19	29.95	29.5	Curcumin + zinc + lifestyle modification	500	zinc + lifestyle modification
Mokhtari et al. 2021	Iran	R, DB, PC, Parallel	Diabetic foot ulcer	B	25	25	12	57.4	55.8	27.5	30.2	Nano‐curcumin	80	Placebo
Neta et al. 2021	Brazil	R, DB, PC, Parallel	T2D	B	33	28	16	63.1	61.9	29.6	28.5	Curcumin + piperine	500	Placebo (carboxymethyl cellulose)
Zamani and Rezagholizadeh 2021 (a)	Iran	R, Open, CO, Parallel	Middle‐aged women with T2D	F	10	10	8	47.2	47.3	31.48	30.6	Curcumin	80	Nothing
Zamani and Rezagholizadeh 2021 (b)	Iran	R, Open, CO, Parallel	Middle‐aged women with T2D	F	10	10	8	46.4	43.5	29.58	30.68	Curcumin + training	80	Training
Asghari et al. 2024 (a)	Iran	R, DB, PC, Parallel	T2D	B	24	23	12	54.56	57.48	27.78	27.82	Curcumin	80	Placebo (oral paraffin)
Asghari et al. 2024 (b)	Iran	R, DB, PC, Parallel	T2D	B	25	23	12	56.68	56.88	28.01	28.9	Curcumin + EPA	80	Placebo (oral paraffin) + EPA
Hosseini et al. 2024	Iran	R, DB, PC, Parallel	T2D and hypertriglyceridemia	B	33	32	12	55.25	56.17	31.55	30.55	Curcumin + piperine	500	Placebo (maltodextrin)
Uchio et al. 2024	Japan	R, DB, PC, Parallel	Overweight and prediabetes	B	39	42	12	55.1	54.7	26.1	26	CLE ( *Curcuma longa* extract)	970	Placebo
Yaikwawong et al. 2024	Thailand	R, DB, PC, Parallel	Obese patients with T2D	B	114	115	48	NR	NR	27.21	26.76	Curcumin	1500	Placebo
El‐Rakabawy et al. 2025	Egypt	R, Open, CO, Parallel	T2D with ASCVD	B	36	36	14	59.8	60.9	35.1	36.1	Curcumin + conventional therapy	1500	Conventional therapy
Ghazaee et al. 2025	Iran	R, TB, PC, Parallel	Prediabetic	B	26	30	12	48.03	48.76	27.94	26.69	Curcumin + piperine	500	Placebo (microcrystalline cellulose)
Lamichhane et al. 2025	USA	R, DB, PC, Parallel	Prediabetic Older Adults	B	14	9	12	65.5	67	32.36	29.52	Curcumin	80	Placebo (dextrin)
Mansour et al. 2025	Iran	R, DB, PC, Parallel	T2D	B	41	45	16	62.32	62.67	29.554	28.174	Nano‐curcumin	80	Placebo
Nada et al. 2025	Egypt	R, DB, PC, Parallel	T2D	B	20	20	12	57.4	56.15	33.58	31.92	Curcumin + black pepper + Glimepiride	1100	Placebo + Glimepiride
Yaikwawong et al. 2025	Thailand	R, DB, PC, Parallel	T2D and MASLD	B	39	39	48	57.33	60.28	27.24	27.5	Curcumin	1500	Placebo

Abbreviations: ASCVD, Atherosclerotic Cardiovascular Disease; BMI, Body Mass Index; CG, control group; CKD, Chronic Kidney Disease; CO, controlled; DB, double‐blinded; EPA, Eicosapentaenoic acid; FPG, Fasting Plasma Glucose; HD, Hemodialysis; IG, intervention group; MASLD, Metabolic dysfunction‐associated steatotic liver disease; NIDDM, Non‐Insulin Dependent Diabetes Mellitus; NR, not reported; PC, placebo‐controlled; R, randomized; SB, single‐blinded; T2D, Type 2 Diabetes; TB, triple‐blinded.

### 3.3 Risk of Bias Assessment

**FIGURE 2:**
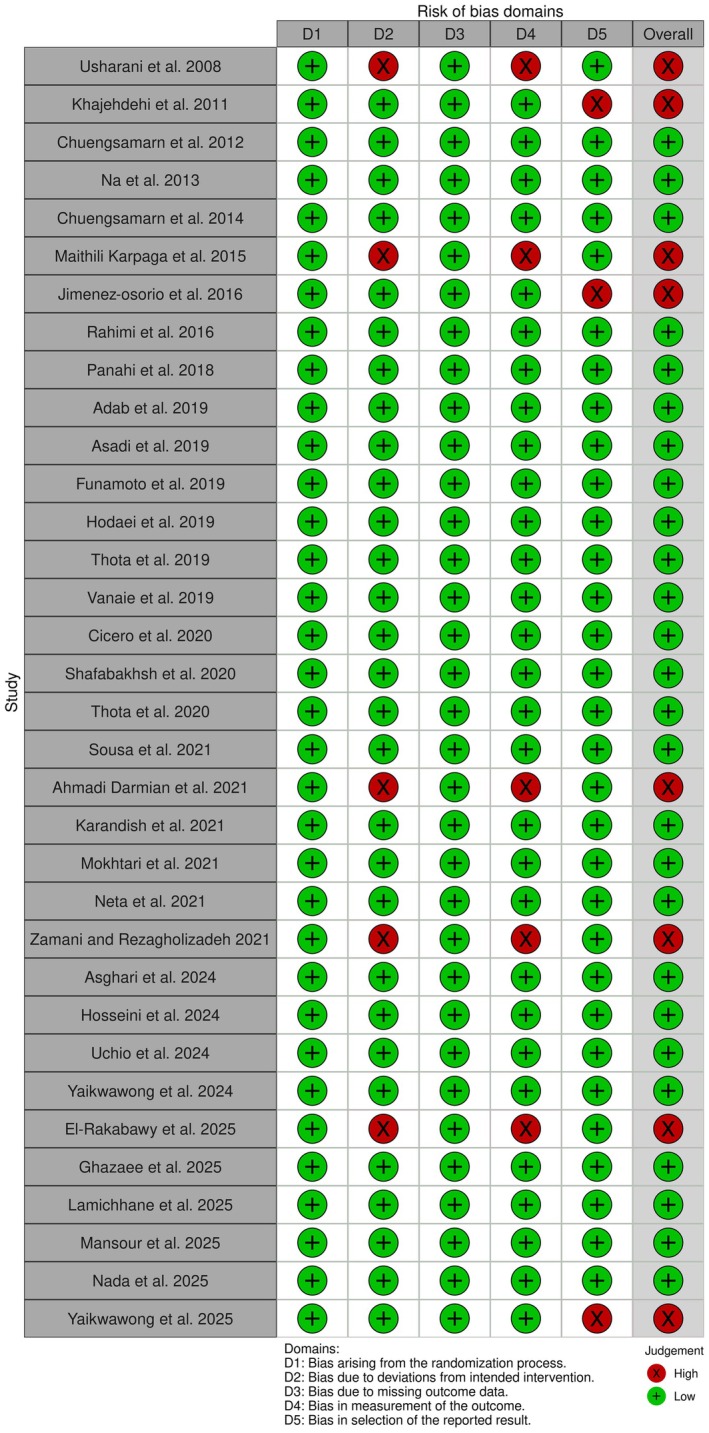
Results of risk of bias evaluation according to the Cochrane tool.

Risk of bias assessment that was performed using the ROB 2 tool identified a general risk of bias as high for eight included studies (Darmian et al. 2021; El‐Rakabawy et al. 2025; Jiménez‐Osorio et al. 2016; Khajehdehi et al. 2011; Maithili Karpaga Selvi et al. 2015; Usharani et al. 2008; Yaikwawong et al. 2025; Zamani and Rezagholizadeh 2021), while the rest had a low general risk of bias. Details of the risks of bias assessment in each domain of the ROB 2 tool are exhibited in Figure 2.

### 3.4 Meta‐Analysis

#### 3.4.1 Effect of Curcumin/Turmeric Intake on FBG Levels

**FIGURE 3:**
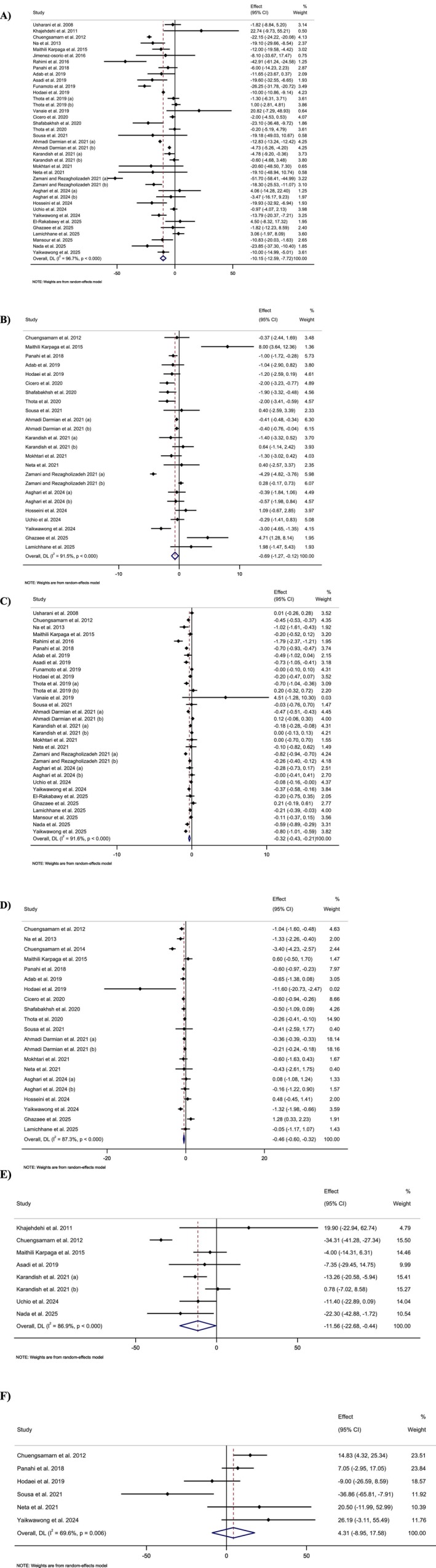
Forest plot detailing weighted mean difference and 95% confidence intervals (CIs) for the effect of curcumin/turmeric on (A) fasting blood glucose (mg/dl), (B) Insulin (μU/ml), (C) Glycated hemoglobin (HbA1c) (%), (D) HOMA‐IR, (E) OGTT (mg/dl), and (F) HOMA‐B.

Pooling 38 effect sizes showed that curcumin/turmeric supplementation led to a significant reduction in FBG (WMD = −10.15 mg/dL; 95% CI, −12.59 to −7.72; *p* < 0.001) (Figure 3A). However, there was a significant heterogeneity across the included effect sizes (*I* ^2^ = 96.7%, *p* = < 0.001). Furthermore, subgroup analysis, performed to find the possible sources of heterogeneity, demonstrated that curcumin/turmeric intake had no significant effects on FBG levels in studies on participants with prediabetes or in those with a normal BMI (Table 2).

**TABLE 2:** Subgroup analyses of curcumin/turmeric on glycemic control markers in prediabetes and diabetes.

	Number of effect sizes	WMD (95% CI)	*p*‐value	Heterogeneity		
				*p* heterogeneity	*I* ^2^	*p* between sub‐groups
*Curcumin intake on FBG*						
Overall effect	38	−10.15 (−12.59, −7.72)	**< 0.001**	< 0.001	96.7%	
Location of study						
Iran	20	−12.26 (−15.53, −9.00)	**< 0.001**	< 0.001	97.6%	0.265
Non‐Iran	18	−8.61 (−14.13, −3.09)	**0.002**	< 0.001	95.0%	
Trial duration (week)						
≥ 12	26	−9.29 (−14.07, −4.52)	**< 0.001**	< 0.001	92.9%	0.254
< 12	12	−12.86 (−16.68, −9.04)	**< 0.001**	< 0.001	98.6%	
Intervention dose (g/day)						
≥ 1	15	−8.19 (−11.48, −4.90)	**< 0.001**	< 0.001	98.2%	0.166
< 1	23	−12.85 (−18.56, −7.14)	**< 0.001**	< 0.001	93.2%	
Intervention type						
Unformulated curcumin	20	−7.01 (−9.96, −4.06)	**< 0.001**	< 0.001	97.6%	0.108
Formulated curcumin	11	−18.84 (−29.83, −7.86)	**0.001**	< 0.001	96.2%	
Piperine‐enhanced	7	−9.94 (−17.12, −2.76)	**0.007**	0.004	68.1%	
Baseline BMI (kg/m^2^)						
Normal (18.5–24.9)	3	−13.48 (−28.37, 1.40)	0.076	< 0.001	93.3%	0.907
Overweight (25–29.9)	23	−10.20 (−13.08, −7.32)	**< 0.001**	< 0.001	97.3%	
Obese (> 30)	10	−10.96 (−20.95, −0.97)	**0.031**	< 0.001	96.1%	
Health status						
Diabetes	28	−14.19 (−17.07, −11.30)	**< 0.001**	< 0.001	96.6%	0.003
Prediabetes	10	−3.06 (−9.72, 3.60)	0.367	< 0.001	96.9%	
*Curcumin intake on Insulin*						
Overall effect	24	−0.69 (−1.27, −0.12)	**0.019**	< 0.001	91.5%	
Location of study						
Iran	15	−0.74 (−1.42, −0.05)	**0.035**	< 0.001	94.0%	0.614
Non‐Iran	9	−0.36 (−1.67, 0.96)	0.595	< 0.001	75.7%	
Trial duration (week)						
≥ 12	16	−0.58 (−1.22, 0.05)	0.074	0.003	57.0%	0.593
< 12	8	−0.91 (−1.92, 0.10)	0.079	< 0.001	97.0%	
Intervention dose (g/day)						
≥ 1	8	−0.79 (−1.38, −0.21)	**0.008**	< 0.001	76.8%	0.592
< 1	16	−0.45 (−1.54, 0.63)	0.415	< 0.001	92.7%	
Intervention type						
Unformulated curcumin	13	−0.50 (−0.93, −0.06)	**0.023**	0.003	59.6%	0.382
Formulated curcumin	5	−1.84 (−4.23, 0.53)	0.129	< 0.001	97.6%	
Piperine‐enhanced	6	0.11 (−1.31, 1.52)	0.883	0.002	74.4%	
Baseline BMI (kg/m^2^)						
Overweight (25–29.9)	18	−0.59 (−0.94, −0.24)	**0.001**	< 0.001	62.3%	0.001
Obese (> 30)	5	−1.14 (−3.54, 1.24)	0.347	< 0.001	92.5%	
Health status						
Diabetes	16	−0.81 (−1.51, −0.11)	**0.023**	< 0.001	93.9%	0.486
Prediabetes	8	−0.33 (−1.47, 0.79)	0.559	0.002	69.1%	
*Curcumin intake on HbA1c*						
Overall effect	32	−0.32 (−0.43, −0.21)	**< 0.001**	< 0.001	91.6%	
Location of study						
Iran	17	−0.33 (−0.49, −0.17)	**< 0.001**	< 0.001	92.5%	0.837
Non‐Iran	15	−0.31 (−0.46, −0.15)	**< 0.001**	< 0.001	87.9%	
Trial duration (week)						
≥ 12	23	−0.31 (−0.44, −0.18)	**< 0.001**	< 0.001	87.8%	0.847
< 12	9	−0.33 (−0.52, −0.14)	**0.001**	< 0.001	92.4%	
Intervention dose (g/day)						
≥ 1	13	−0.37 (−0.50, −0.23)	**< 0.001**	< 0.001	83.0%	0.50
< 1	19	−0.29 (−0.45, −0.14)	**< 0.001**	< 0.001	91.5%	
Intervention type						
Unformulated curcumin	18	−0.25 (−0.38, −0.13)	**< 0.001**	< 0.001	91.4%	0.499
Formulated curcumin	9	−0.45 (−0.76, −0.15)	**0.004**	< 0.001	94.7%	
Piperine‐enhanced	5	−0.29 (−0.68, 0.08)	0.131	0.001	77.8%	
Baseline BMI (kg/m^2^)						
Normal (18.5–24.9)	3	−0.01 (−0.10, 0.07)	0.753	0.499	0.0%	0.001
Overweight (25–29.9)	20	−0.32 (−0.45, −0.18)	**< 0.001**	< 0.001	91.3%	
Obese (> 30)	8	−0.43 (−0.69, −0.16)	**0.001**	< 0.001	91.8%	
Health status						
Diabetes	24	−0.38 (−0.52, −0.24)	**< 0.001**	< 0.001	90.4%	0.059
Prediabetes	8	−0.18 (−0.33, −0.02)	**0.027**	< 0.001	89.5%	
*Curcumin intake on HOMA‐IR*						
Overall effect	21	−0.46 (−0.60, −0.32)	**< 0.001**	< 0.001	87.3%	
Location of study						
Iran	11	−0.29 (−0.43, −0.15)	**< 0.001**	< 0.001	86.7%	0.039
Non‐Iran	10	−0.89 (−1.44, −0.34)	**0.002**	< 0.001	87.8%	
Trial duration (week)						
≥ 12	15	−0.59 (−1.02, −0.16)	**0.007**	< 0.001	84.1%	0.241
< 12	6	−0.32 (−0.46, −0.18)	**< 0.001**	< 0.001	92.2%	
Intervention dose (g/day)						
≥ 1	9	−0.49 (−0.66, −0.32)	**< 0.001**	< 0.001	93.9%	0.314
< 1	12	−0.29 (−0.64, 0.04)	0.088	0.017	52.3%	
Intervention type						
Unformulated curcumin	12	−0.56 (−0.74, −0.38)	**< 0.001**	< 0.001	92.0%	0.039
Formulated curcumin	3	−0.28 (−0.42, −0.13)	**< 0.001**	0.609	0.0%	
Piperine‐enhanced	6	−0.08 (−0.66, 0.50)	0.788	0.003	71.9%	
Baseline BMI (kg/m^2^)						
Overweight (25–29.9)	17	−0.55 (−0.71, −0.39)	**< 0.001**	< 0.001	89.5%	0.017
Obese (> 30)	3	−0.15 (−0.49, 0.18)	0.382	0.292	18.8%	
Health status						
Diabetes	16	−0.50 (−0.67, −0.34)	**< 0.001**	< 0.001	89.0%	0.381
Prediabetes	5	−0.28 (−0.75, 0.19)	0.243	< 0.001	80.6%	
*Curcumin intake on OGTT*						
Overall effect	8	−11.56 (−22.68, −0.44)	**0.042**	< 0.001	86.9%	
*Curcumin intake on HOMA‐B*						
Overall effect	6	4.31 (−8.95, 17.57)	0.524	0.006	69.6%	

Abbreviations: BMI, body mass index; CI, confidence interval; FBG, fasting blood glucose; HbA1c, hemoglobin A1C; HOMA‐B, homeostasis model assessment of *β*‐cell function; HOMA‐IR, Homeostatic Model Assessment of Insulin Resistance; OGTT, oral glucose tolerance test; WMD, weighted mean differences. Bold indicates statistical significance value (*p* < 0.05).

#### 3.4.2 Effect of Curcumin/Turmeric Intake on Insulin Levels

Merging 24 effect sizes revealed a significant reduction in insulin levels followed by curcumin/turmeric supplementation (WMD = −0.69 μU/ml; 95% CI, −1.27 to −0.12; *p* < 0.001) (Figure 3B). In addition, a significant heterogeneity was observed among the merged effect sizes (*I* ^2^ = 91.5%, *p* < 0.001). Subgroup analysis showed that curcumin/turmeric supplementation led to a significant decrease in insulin levels in studies that were conducted in Iran, with intervention dosage ≥ 1 g/day, or intervention type of unformulated curcumin, similar to trials that were conducted on individuals with T2D or overweight (Table 2).

#### 3.4.3 Effect of Curcumin/Turmeric Intake on HbA1c

Combining 32 effect sizes showed that curcumin/turmeric intake significantly reduced HbA1c (WMD = −0.32%; 95% CI, −0.43 to −0.21; *p* < 0.001) (Figure 3C). Also, there was significant heterogeneity across the included effect sizes (*I* ^2^ = 91.6%, *p* < 0.001). Furthermore, subgroup analysis showed that curcumin/turmeric supplementation had no significant impact on HbA1c in studies that used curcumin combined with Piperine as an intervention or those that involved individuals with normal BMI (Table 2).

#### 3.4.4 Effect of Curcumin/Turmeric Intake on HOMA‐IR

Pooling 21 effect sizes revealed that curcumin/turmeric supplementation led to a significant reduction in HOMA‐IR (WMD = −0.46; 95% CI, −0.60 to −0.32; *p* < 0.001) (Figure 3D). Furthermore, a significant heterogeneity was observed among the combined effect sizes (*I* ^2^ = 87.3%, *p* < 0.001). Subgroup analysis showed that curcumin/turmeric intake had no significant impacts on HOMA‐IR in studies that performed intervention with < 1 g/day dosage or those that used curcumin combined with piperine as intervention, similar to trials that were conducted on individuals with prediabetes or obesity (Table 2).

#### 3.4.5 Effect of Curcumin/Turmeric Intake on OGTT

Meta‐analyzing eight effect sizes demonstrated a significant reduction in OGTT levels due to curcumin/turmeric intake (WMD = −11.53 mg/dL, 95% CI, −22.68 to −0.44; *p* = 0.04) (Figure 3E). However, a significant heterogeneity was detected among the effect sizes that were pooled (*I* ^2^ = 86.9%, *p* < 0.001).

#### 3.4.6 Effect of Curcumin/Turmeric Intake on HOMA‐B

Combining six effect sizes showed no significant alteration in HOMA‐B followed by curcumin/turmeric intake (WMD = 4.31; 95% CI, −8.95 to 17.57; *p* = 0.52) (Figure 3F). Also, there was significant heterogeneity among the included effect sizes (*I* ^2^ = 69.6%, *p* = 0.006).

### 3.5 Meta Regression and Dose Response Analyses

Meta regression analysis showed no significant linear relationship between the characteristics of intervention, including dosage and duration, and changes in FBG, HbA1c, insulin, or HOMA‐IR (*p* > 0.05) (Figures S1A–D and 2A–D). Furthermore, dose–response analysis that was performed using the fractional polynomial modeling revealed a significant non‐linear association between dosage of intervention and changes in FBG (coefficient: 75.35, *p*‐value = 0.01) or HbA1c levels (coefficient: 1.39, *p*‐value = 0.009). However, there was no significant non‐linear relationship between the dosage of intervention and insulin or HOMA‐IR level changes (*p* > 0.05). Furthermore, a significant non‐linear association was observed between the durations of intervention and insulin level changes (coefficient: −1.87, *p*‐value = 0.04). Meanwhile, the durations of intervention and changes in FBG, HbA1c, or HOMA‐IR levels (*p* > 0.05) (Figure S3A–H).

### 3.6 Sensitivity Analysis

Sensitivity analysis showed that the overall effect size of the effect of curcumin/turmeric intake on FBG, HbA1c, or HOMA‐IR or HOMA‐B was not significantly dependent on one specific included effect size. However, the pooled effect size for insulin was significantly changed, followed by the omission of the Darmian et al. (2021) (a) (WMD: −0.56, 95% CI: −1.34, 0.21), or Darmian et al. (2021) (b) (WMD: −0.63, 95% CI: −1.31, 0.04) (Darmian et al. 2021). Also, the overall effect size for OGTT was significantly changed, followed by excluding the Asadi et al. (2019) (WMD: −11.97 mg/dL, 95% CI: −23.95, 0.00) (Asadi et al. 2019); Karandish et al. (2021) (a) (WMD: −10.88 mg/dL, 95% CI: −24.84, 3.06) (Karandish et al. 2021); Uchio et al. (2024) (WMD: −11.41 mg/dL, 95% CI: −24.22, 1.39) (Uchio et al. 2024); or Nada et al. (2025) (WMD: −10.22 mg/dL, 95% CI: −22.25, 1.81) (Nada et al. 2025).

### 3.7 Publication Bias

Egger regression test, similar to visual inspection of funnel plots, indicated no significant publication bias among the studies that assessed the impacts of curcumin/turmeric supplementation on FBG (*p*_Egger_ = 0.87), insulin (*p*_Egger_ = 0.48), HbA1c (*p*_Egger_ = 0.41), OGGT (*p*_Egger_ = 0.19), HOMA‐IR (*p*_Egger_ = 0.50), or HOMA‐B (*p*_Egger_ = 0.58) (Figure S4A–F).

### 3.8 Certainty Assessment

The certainty assessment was performed according to the GRADE framework. The levels of certainty for the impacts of curcumin/turmeric on HOMA‐B were identified as moderate due to serious inconsistency. Furthermore, the level of evidence for FBG, Insulin, HbA1c, HOMA‐IR, or OGTT was downgraded to low due to very serious inconsistency. The details of the limitation assessment for evidence are exhibited in Table S2.

## 4 Discussion

This systematic review and meta‐analysis of 34 randomized controlled trials provides a comprehensive and updated synthesis of the effects of curcumin/turmeric supplementation on markers of glycemic control in individuals with prediabetes and type 2 diabetes. The main findings indicate that curcumin/turmeric supplementation leads to a significant improvement in key glycemic parameters, including FBG, HbA1c, fasting insulin, HOMA‐IR, and OGTT, compared to control groups. However, no significant changes were observed in HOMA‐B levels following this intervention. Importantly, these benefits were accompanied by very high heterogeneity. The pooled analysis demonstrated that curcumin/turmeric supplementation significantly reduced FBG (WMD = −10.15 mg/dL) and HbA1c (WMD = −0.32%). Although modest, these reductions are potentially clinically relevant when curcumin is considered as an adjunct therapy rather than a replacement for conventional treatment. A 1% reduction in HbA1c is associated with a 37% reduction in risk for microvascular complications and a 21% reduction in diabetes‐related mortality (Arnold et al. 2022; Sartore et al. 2023). Our observed HbA1c reduction of −0.32% is comparable to some nutraceutical interventions and approaches the lower end of effects seen with first‐line pharmacological agents such as metformin (−0.5% to −1.0%) in high‐risk individuals (Knowler et al. 2002). Furthermore, an FBG reduction of ~10 mg/dL aligns with the threshold considered clinically meaningful for mitigating diabetes‐related risks (Association, A. D 2022). This suggests curcumin could be considered a valuable complementary strategy in the management of hyperglycemia.

Our findings are further contextualized by a series of recent meta‐analyses, which, while often including fewer studies, report both congruent and divergent results. Consistent with our primary glycemic findings, Tian et al. (Tian et al. 2022) and Mokgalaboni et al. (Mokgalaboni et al. 2024) also reported significant reductions in FBG (WMD = −8.85 mg/dL and −11.48 mg/dL, respectively) and HbA1c (WMD = −0.54% for both) in T2D populations. The slightly larger HbA1c effect size in these analyses compared to ours (−0.32%) may be attributed to their exclusive focus on T2D, whereas our inclusion of prediabetic studies, which showed milder effects, likely diluted the overall pooled estimate. This theory is supported by the results of Altobelli et al. (Altobelli et al. 2021), whose analysis of uncomplicated T2D found no significant effect on FBG, underscoring the substantial heterogeneity within even narrowly defined populations and the impact of study selection on meta‐analytic conclusions. Notably, our work provides a more comprehensive glycemic profile by investigating the impact of curcumin/turmeric supplementation on fasting insulin, OGTT, HOMA‐IR, and HOMA‐B, while previous meta‐analyses did not include these outcomes in their study.

The significant reductions in fasting insulin (WMD = −0.69 mU/L) and HOMA‐IR (WMD = −0.46) following supplementation with curcumin/turmeric point toward an improvement in insulin sensitivity, which is a cornerstone in the pathophysiology of T2D. This is a critical finding as it suggests curcumin's benefits extend beyond merely lowering circulating glucose to addressing the underlying insulin resistance.

Turmeric is a complex plant with multiple bioactive compounds, each of which may contribute in distinct ways to improving glycemic control (H. A. Zhang and Kitts 2021). The main group of turmeric is identified as curcuminoids, including curcumin, demethoxycurcumin, and bisdemethoxycurcumin (M. T. El‐Saadony et al. 2025). Curcuminoids have been shown to decrease insulin and glucose levels and improve insulin resistance (Marton, Pescinini‐e‐Salzedas, et al. 2021; Marton, Pescinini, et al. 2021). In addition, curcumin's anti‐diabetic features may be related to its ability to reduce inflammation and oxidative stress (Marton, Pescinini‐e‐Salzedas, et al. 2021; Marton, Pescinini, et al. 2021). Beyond curcuminoids, turmeric volatile oils are more effective than the reference standard drug acarbose in the inhibition of glucosidase enzyme (Lekshmi et al. 2012). Curcumin may exert antidiabetic effects through multiple mechanisms, such as suppressing the gluconeogenesis by activating hepatic AMP‐activated protein kinase (AMPK) (Kim et al. 2009), promote glucose uptake by enhancing the expression of Glucose transporter type 4 (GLUT‐4) in skeletal muscle through the PLC‐PI3K pathway (Zhang et al. 2013), and reducing oxidative stress markers, which may improve insulin sensitivity and support *β*‐cell preservation (Roney and Mohd Aluwi 2025).

The lack of a significant effect on HOMA‐B indicates that the primary mechanism of action in these studies may not be a direct restoration of pancreatic beta‐cell function but rather an enhancement of insulin action in peripheral tissues. This is consistent with preclinical evidence showing curcumin's ability to activate AMPK, suppress nuclear factor kappa‐light‐chain‐enhancer of activated B cells (NF‐κB)‐mediated inflammation, and inhibit tumor necrosis factor alpha (TNF‐*α*), all of which improve insulin signaling (H.‐J. He et al. 2012; Jiménez‐Flores et al. 2014). Additionally, curcumin may ameliorate insulin resistance by reducing adipose tissue inflammation and inhibiting key enzymes like protein kinase C (PKC) and thioredoxin‐interacting protein (TXNI), which are implicated in impaired insulin receptor signaling (Y. He et al. 2015; Shao et al. 2012).

Heterogeneity was consistently high (*I* ^2^ > 90% for most outcomes). As shown by subgroup analyses, the observed heterogeneity across studies can largely be explained by differences in participant characteristics (e.g., baseline BMI and health status), the type of curcumin formulation, and the geographical location of the studies. For instance, the health status of the population was a significant effect modifier. The beneficial effects on FBG, insulin, and HOMA‐IR were more pronounced and consistent in studies conducted on individuals with T2D compared to those with prediabetes, where effects were often nullified. This may be because individuals with overt diabetes have a greater margin for improvement in their glycemic parameters, whereas those with prediabetes may have milder dysregulation that is less responsive to intervention or may require longer duration studies to see an effect.

Also, the formulation and dosage of the intervention played a critical role. The subgroup analysis for insulin showed that significant reductions were achieved with dosages ≥ 1 g/day and with unformulated curcumin, but not with lower doses or with curcumin combined with piperine. This was an unexpected finding, as piperine is added to enhance bioavailability (Shoba et al. 1998). Similarly, for HOMA‐IR, formulations combining curcumin with piperine showed no significant effect. This paradox may be due to the specific study populations, the specific ratios of curcumin to piperine used, or other unknown interactions in these particular trials. It is plausible that while piperine enhances absorption, it may also influence the metabolism of curcumin or interact with other pathways in a way that modulates its glucoregulatory effects, an area requiring further pharmacokinetic study (Prasad et al. 2014). The results of our non‐linear dose–response analysis, which showed a significant relationship between dosage and reductions in FBG and HbA1c, further underscore the importance of optimal dosing. The finding that longer duration was associated with greater reductions in insulin levels suggests that the benefits on insulin sensitivity may be cumulative over time.

Finally, the baseline BMI of participants influenced the results. Interventions had no significant impact on FBG or HbA1c in populations with a normal BMI, suggesting that the glucoregulatory benefits of curcumin may be particularly relevant in the context of overweight and obesity, conditions intrinsically linked to inflammation and insulin resistance. This supports the notion that curcumin's efficacy is closely tied to the presence of metabolic dysfunction and underlying inflammation, which are more prevalent in higher BMI categories (Bullon et al. 2014).

To our best knowledge, the present review is the most comprehensive systematic review and meta‐analysis that investigates the impact of curcumin/turmeric supplementation on glycemic outcomes that involve both individuals with prediabetes and T2D. Performing risk of bias assessment based on the most up‐to‐date recommendation tool for risk of bias assessment by the Cochrane Working Group, performing meta‐regression and dose–response analysis to assess the linear and non‐linear associations of features of supplementation (durations, dosages) with changes in outcomes, respectively, and evaluation of certainty of evidence according to the GRADE framework were other strengths of this review.

## 5 Limitations

This review includes several limitations. First, the high heterogeneity, despite our extensive efforts to explore it, indicates that there are likely other unmeasured factors influencing the results. Second, the risk of bias assessment also identified several studies with a high overall risk of bias, which could affect the effect estimates. Third, the included studies varied widely in their design, comparator groups (placebo vs. active control), and the background medication of participants, which could confound the results. Fourth, the dose–response analysis, while informative, was limited by the number of data points available across the dosage spectrum. Finally, the overall certainty of the evidence for all outcomes was rated as low (for FBG, insulin, HbA1c, HOMA‐IR, OGTT) or moderate (for HOMA‐B) according to the GRADE.

## 6 Implications and Future Research

The findings of this meta‐analysis suggest that curcumin/turmeric supplementation can be a beneficial adjunct therapy for improving glycemic control, particularly in individuals with T2D who are overweight or obese. Clinicians may need to take the formulation and dosage into account, as some evidence suggests potential benefits from unformulated curcumin/curcuminoids or nano‐curcumin at doses of ≥ 1 g/day for a duration of at least 8–12 weeks. However, definitive conclusions regarding the clinical application of curcumin/turmeric supplementation in the management of T2D need to be based on more high‐quality evidence being available.

Future research should prioritize large‐scale, well‐designed RCTs that directly compare different bioavailable formulations (e.g., nano‐curcumin vs. curcumin with piperine) to establish superiority. Studies should be of longer duration to assess the sustainability of the effect and should specifically target prediabetic populations to determine if curcumin can effectively delay the progression to T2D. Furthermore, future trials should standardize reporting and strive to measure and report on all glycemic parameters consistently to allow for more robust pooling of data.

## 7 Conclusion

In conclusion, this meta‐analysis provides evidence that curcumin/turmeric supplementation significantly improves key markers of glycemic control and insulin resistance in individuals with T2D and prediabetes. However, no significant alteration in HOMA‐B was observed following supplementation with curcumin/turmeric. Importantly, the effects are inconsistent and influenced by populations, dosage, and formulation. Despite the promising evidence, findings should be interpreted with caution due to significant heterogeneity that led to downgraded certainty of evidence. Therefore, curcumin may be considered as an adjunct to standard lifestyle and pharmacological therapies for glycemic management, but not as a replacement. Further high‐quality, long‐term trials are needed to solidify these findings and translate them into definitive clinical recommendations.

## Author Contributions

**Haniyeh Golafrouz:** investigation, data curation. **Hossein Bahari:** conceptualization, writing – original draft, writing – review and editing, methodology, formal analysis, project administration, validation. **Mostafa Shahraki Jazinaki:** writing – original draft, writing – review and editing, software.

## Funding

The authors have nothing to report.

## Conflicts of Interest

The authors declare no conflicts of interest.

## Supporting information


**Figure S1:** Random‐effects meta‐regression plots of the association between mean changes in (A) fasting blood glucose (mg/dl), (B) Insulin (μIU/ml), (C) Glycated hemoglobin (HbA1c) (%), (D) HOMA‐IR, and curcumin/turmeric dosage (mg/day).
**Figure S2:** Random‐effects meta‐regression plots of the association between mean changes in (A) fasting blood glucose (mg/dl), (B) Insulin (μIU/ml), (C) Glycated hemoglobin (HbA1c) (%), (D) HOMA‐IR, and intervention duration (weeks).
**Figure S3:** Dose–response relations between dosage (mg/day) and duration (weeks) of curcumin/turmeric supplementation and mean difference in fasting blood glucose (A, B), Insulin (C, D), Glycated hemoglobin (HbA1c) (E, F), and HOMA‐IR (G, H).
**Figure S4:** Funnel plots for the effect of curcumin/turmeric on (A) fasting blood glucose (mg/dl), (B) Insulin (μU/ml), (C) Glycated hemoglobin (HbA1c) (%), (D) HOMA‐IR, (E) OGTT (mg/dl), and (F) HOMA‐B.

**Table S1:** Search strategy.

**Table S2:** GRADE profile of curcumin/turmeric on glycemic control in prediabetes and diabetes.

## Data Availability

All data generated or analyzed during this study are included in this published article.
