# Supplementary material for: Curcumin/Turmeric Supplementation on Glycemic Control in Adults With Prediabetes and Type 2 Diabetes: A Systematic Review and Dose–Response Meta‐Analysis
Source: Food Sci Nutr. 2026 Apr 16;14(4):e71748. doi: 10.1002/fsn3.71748 (PMC13087110; doi:10.1002/fsn3.71748)
Supplement: Supplementary file 1 — Figure S1: Random‐effects meta‐regression plots of the association between mean changes in (A) fasting blood glucose (mg/dl), (B) Insulin (μIU/ml), (C) Glycated hemoglobin (HbA1c) (%), (D) HOMA‐IR, and curcumin/turmeric dosage (mg/day). Figure S2: Random‐effects meta‐regression plots of the association between mean changes in (A) fasting blood glucose (mg/dl), (B) Insulin (μIU/ml), (C) Glycated hemoglobin (HbA1c) (%), (D) HOMA‐IR, and intervention duration (weeks). Figure S3: Dose–response relations between dosage (mg/day) and duration (weeks) of curcumin/turmeric supplementation and mean difference in fasting blood glucose (A, B), Insulin (C, D), Glycated hemoglobin (HbA1c) (E, F), and HOMA‐IR (G, H). Figure S4: Funnel plots for the effect of curcumin/turmeric on (A) fasting blood glucose (mg/dl), (B) Insulin (μU/ml), (C) Glycated hemoglobin (HbA1c) (%), (D) HOMA‐IR, (E) OGTT (mg/dl), and (F) HOMA‐B. [file FSN3-14-e71748-s003.docx]

**Supplementary Figures**

**A)**

**B)**

**C)**

**D)**

**Supplementary Figure 1.** Random-effects meta-regression plots of the association between mean changes in A) fasting blood glucose (mg/dl), B) Insulin (μIU/ml), C) Glycated hemoglobin (HbA1c) (%), D) HOMA-IR, and curcumin/turmeric dosage (mg/day).

**A)**

**B)**

**C)**

**D)**

**Supplementary Figure 2.** Random-effects meta-regression plots of the association between mean changes in A) fasting blood glucose (mg/dl), B) Insulin (μIU/ml), C) Glycated hemoglobin (HbA1c) (%), D) HOMA-IR, and intervention duration (weeks).

**A)**

**B)**

**C)**

**D)**

**E)**

**F)**

**G)**

**H)**

**Supplementary Figure 3.** Dose-response relations between dosage (mg/day) and duration (weeks) of curcumin/turmeric supplementation and mean difference in fasting blood glucose (A, B), Insulin (C, D), Glycated hemoglobin (HbA1c) (E, F), and HOMA-IR (G, H).

**A)**

**B)**

**C)**

**D)**

**E)**

**F)**

**Supplementary Figure 4.** Funnel plots for the effect of curcumin/turmeric on A) fasting blood glucose (mg/dl), B) Insulin (μU/ml), C) Glycated hemoglobin (HbA1c) (%), D) HOMA-IR, E) OGTT (mg/dl), and F) HOMA-B.
