# Supplementary material for: Curcumin/Turmeric Supplementation on Glycemic Control in Adults With Prediabetes and Type 2 Diabetes: A Systematic Review and Dose–Response Meta‐Analysis
Source: Food Sci Nutr. 2026 Apr 16;14(4):e71748. doi: 10.1002/fsn3.71748 (PMC13087110; doi:10.1002/fsn3.71748)
Supplement: Supplementary file 3 — Table S2: GRADE profile of curcumin/turmeric on glycemic control in prediabetes and diabetes. [file FSN3-14-e71748-s001.docx]

**Supplementary Table 2.** GRADE profile of Curcumin/Turmeric on glycemic control in prediabetes and diabetes.

| **Outcomes** | **Risk of bias** | **Inconsistency** | **Indirectness** | **Imprecision** | **Publication Bias** | **Quality**  **of evidence** |
| --- | --- | --- | --- | --- | --- | --- |
| FBG | No serious limitation | Very serious limitation ^1^ | No serious limitation | No serious limitation | No serious limitation | ⊕⊕◯◯  Low |
| Insulin | No serious limitation | Very serious limitation | No serious limitation | No serious limitation | No serious limitation | ⊕⊕◯◯  Low |
| HbA1c | No serious limitation | Very serious limitation | No serious limitation | No serious limitation | No serious limitation | ⊕⊕◯◯  Low |
| HOMA-IR | No serious limitation | Very serious limitation | No serious limitation | No serious limitation | No serious limitation | ⊕⊕◯◯  Low |
| OGTT | No serious limitation | Very serious limitation | No serious limitation | No serious limitation | No serious limitation | ⊕⊕◯◯  Low |
| HOMA-B | No serious limitation | Serious limitation ^2^ | No serious limitation | No serious limitation | No serious limitation | ⊕⊕⊕◯  Moderate |

1. There is high heterogeneity (I^2^>75%)
2. There is high heterogeneity (I^2^>50%)
